# Genomic-Based Optimum Contribution in Conservation and Genetic Improvement Programs with Antagonistic Fitness and Productivity Traits

**DOI:** 10.3389/fgene.2016.00025

**Published:** 2016-02-24

**Authors:** Enrique Sánchez-Molano, Ricardo Pong-Wong, Georgios Banos

**Affiliations:** ^1^Division of Genetics and Genomics, The Roslin Institute and Royal (Dick) School of Veterinary Studies, University of EdinburghEdinburgh, UK; ^2^Scotland’s Rural CollegeEdinburgh, UK; ^3^School of Veterinary Medicine, Aristotle University of ThessalonikiThessaloniki, Greece

**Keywords:** optimum contribution, inbreeding, genomic selection, fitness, productivity

## Abstract

Animal selection for genetic improvement of productivity may lead to an increase in inbreeding through the use of techniques that enhance the reproductive capability of selected animals. Therefore, breeding strategies aim to balance maintaining genetic variability and acceptable fitness levels with increasing productivity. The present study demonstrates the effectiveness of genomic-based optimum contribution strategies at addressing this objective when fitness and productivity are genetically antagonistic traits. Strategies are evaluated in directional selection (increasing productivity) or conservation (maintaining fitness) scenarios. In the former case, substantial rates of genetic gain can be achieved while greatly constraining the rate of increase in inbreeding. Under a conservation approach, inbreeding depression can be effectively halted while also achieving a modest rate of genetic gain for productivity. Furthermore, the use of optimum contribution strategies when combined with a simple non-random mating scheme (minimum kinship method) showed an additional delay in the increase of inbreeding in the short term. In conclusion, genomic-based optimum contribution methods can be effectively used to control inbreeding and inbreeding depression, and still allow genetic gain for productivity traits even when fitness and productivity are antagonistically correlated.

## Introduction

Over the past 30 years, selective breeding has mainly focused on production traits, with some of these traits being dramatically improved (Hayes et al., 2013). However, new issues and challenges have recently arisen as a consequence of increased concern for biodiversity, animal robustness, welfare, and market preferences toward product hygiene and quality (Olynk, 2012), thus requiring the re-assessment of strategies to address the new objectives.

With previous selection pressure being focused mainly on production, the genetic variability of many functional traits (e.g., fertility) has been eroded as a consequence of the negative antagonistic correlation with productivity traits and the lack of selection pressure to improve them (Hoekstra et al., 1994; Pryce et al., 2002; Oltenacu and Broom, 2010). This can be sorted by constructing a selection index to allow for selection for productivity while preventing the fast reduction in fitness (van der Werf et al., 2009). However, the low heritability and lack of relevant data means that low or modest improvements can be achieved, thus rendering necessary alternative strategies such as genomic prediction to increase the accuracy of predictions.

Furthermore, the widespread use of artificial insemination, multiple ovulation and embryo transfer in some species has led to selected parents of high genetic merit having 100s to 10s of 1000s of progeny (Brackett, 2012). This has resulted in a high level of inbreeding, which could be related to a loss in fitness. In order to control the increase in inbreeding resulting from selection, optimum contribution strategies have been developed in livestock genetic improvement schemes to maximize the genetic gain for a pre-set level of inbreeding (Wray and Goddard, 1994; Meuwissen, 1997; Grundy et al., 1998). These methods take into consideration the genetic merit of candidates and their genetic relationships in order to determine the optimum number of progeny for each candidate. Alternative implementations of these strategies focused on conservation programs (i.e., for endangered species) aiming to minimize inbreeding and enhance fitness (Ballou and Lacy, 1995; Fernández et al., 2011). Although these dynamic methods are mainly based on the optimization of candidate selection and subsequent random mating, they can be also combined with non-random mating strategies in two-step programs to achieve a further reduction in inbreeding (Sonesson and Meuwissen, 2000).

Hence, to ensure that the maximum benefit is achieved, selection programs should combine strategies for (i) increasing the accuracy of EBV and (ii) optimizing the selection of candidates and their genetic contributions. Previous studies combining genomic predictions with optimized selection have shown a synergistic effect leading to greater selection response (Nielsen et al., 2011; Pryce et al., 2012; Sonesson et al., 2012; Clark et al., 2013). Furthermore, genomic estimates provide a more precise estimate of the true genetic relationships among animals than the obtained with the traditional pedigree-based relationship matrix (Sonesson et al., 2012). However, so far, no previous study has addressed the dynamics of the above when selection considers two genetically antagonistic traits.

The present study addresses genomic-based optimum contribution in the presence of genetic antagonism between key functional and production traits. Two main scenarios are tested in a simulation study focusing on (i) a genetic improvement scheme aiming at maximizing genetic gain while controlling inbreeding and (ii) a conservation program aiming at minimizing inbreeding while allowing for genetic gains.

## Materials and Methods

### Simulation of Populations

A base population with a size of 2,000 animals (1,000 males and 1,000 females) was simulated with initial allelic frequencies of 0.5 for all loci and randomly mated for 50 generations to allow the establishment of linkage disequilibrium between markers and the QTL following a similar process as in Behmaram et al. (2013) and Boustan et al. (2013). After the 50th generation, 1,000 individuals (500 males and 500 females) were randomly chosen as the base generation of the simulation of the ensuing monitoring period; the latter consisted of 20 generations under different selection and optimum contribution strategies described below.

### Simulation of Genomes

For each animal, the genome consisted of 20 chromosomes of equal length (140 cM), with 64,000 bi-allelic single nucleotide polymorphisms (SNPs) evenly distributed among them (3,200 nucleotides per chromosome). One 1000 SNPs were considered as functional genes and randomly sampled without replacement. In addition, 10,000 SNPs were also randomly chosen without replacement and selected as genetic markers in linkage disequilibrium with the functional genes. These SNPs were used to compute identity-by-state (IBS) genomic relationships among individual animals. Mutation rate was assumed to be 2.2 × 10^-5^ per nucleotide (Brito et al., 2011) and recombination was simulated based on SNP distance using the Haldane mapping function (Haldane, 1919).

### Simulated Traits

Two main traits were considered: (i) a productivity trait with a moderate-high heritability (0.30) and (ii) a fitness-related trait with a low heritability (0.10), reflecting a threshold-based ability of the animal to survive and reproduce. These heritability estimates reflect estimates from studies based on real data in different livestock species (Luan et al., 2009).

Productivity was assumed to be a mainstream trait that will be normally selected for in a livestock genetic improvement program. Fitness was assumed to be an important trait antagonistically related with productivity, which may or may not be included in the selection program, as explained later. The antagonistic genetic correlation between the two traits was assumed to be -0.50, with half of the genes being simulated to have an equal but opposite effect on the two traits and therefore, being representative of a pessimistic scenario considering previous estimates of negative correlations between productivity and fitness (Ingvartsen et al., 2003; Oltenacu and Broom, 2010). Furthermore, fitness was assumed to be affected by inbreeding depression, as explained later.

The phenotypic variance of each trait was standardized to 1 and, therefore, the additive genetic variance (V_α_) was equal to the heritability of the trait. For each trait, the effects of the functional genes were assumed to follow a normal distribution with mean 0 and variance α^2^, α being the average effect of allelic substitution (α = $\alpha =\sqrt{V_{\alpha }/2npq},$ where *n* is the number of loci affecting the trait and *p* and *q* are the allelic frequencies at a starting value of 0.5 (Falconer and Mackay, 1996).

When simulating both traits, two alternatives were considered: (i) fitness phenotypes were assumed to be available (i.e., recorded) in all animals or (ii) only a proportion of animals (20%) having a relevant phenotypic record.

### True and Predicted Breeding Values

True breeding values (TBVs) for each animal and trait were computed from gene effects and allelic frequencies simulated for the correspondent functional genes, with phenotypic values being simulated by adding to the TBV an environmental deviation normally distributed with mean 0 and variance V _e_. Following classic infinitesimal theory (Nadaf et al., 2012), genomic estimated breeding values (GEBVs) were simulated by adding an error term to the TBV. This error term was computed assuming a targeted accuracy *r* of the GEBVs (TBV-GEBV correlation) and a normal distribution *N* [0, (1-*r*^2^)V_α_] for the error term. The use of this approach to simulate GEBVs has been developed and used in previous studies (Dekkers, 2007; Granleese et al., 2015).

Low heritability traits are expected to have lower genomic prediction accuracies when compared to medium-high heritability traits and, in addition, animals with genotypes and phenotypes (training population) are expected to have higher accuracies than animals with genotypes only (Daetwyler et al., 2010). Therefore, accuracies for productivity GEBVs were assumed to be always 0.70, as all animals were simulated to have phenotypic records. Accuracies for fitness were assumed to be 0.50 for animals with phenotypic records and 0.40 for animals without phenotypic records.

### Selection Index

Different combinations of selection on productivity and fitness were considered: (a) index I50 was created as a 50%/50% combination of the productivity and fitness GEBVs (equal emphasis); (b) index I25 was created as a 75%/25% productivity/fitness GEBV combination; and (c) index I0 included only productivity GEBVs. These weights were meant to reflect the relative emphasis placed on each trait, independently of the assumed heritabilities and genetic correlation.

### Inbreeding and Inbreeding Depression

The genomic relationship matrix (G) based on IBS relationships among animals was computed in every generation using the method of Van Raden (2008):

$$G=ZZ^{\prime }/k$$

with Z being the centered matrix (subtraction of the expected genotype frequencies from the incidence matrix with genomic information) and *k* the scaling parameter computed as *k* = 2Σ*pq*, where *p* and *q* are the allelic frequencies at the base generation of the simulation.

Genomic inbreeding for each individual (i) was computed as *G_ii_* – 1, as these inbreeding coefficients represent the correlation between uniting gametes in an individual. Pedigree inbreeding based on pedigree relationships was also computed for comparison, assuming that animals in the base population were unrelated.

As mentioned above, simulated fitness was assumed to be affected by inbreeding depression. Therefore, a phenotypic reduction of 5% in fitness per 0.1 (10%) increase in inbreeding was assumed in concordance with previous studies (Theodorou and Couvet, 2006). For inbreeding depression purposes, only genomic inbreeding was considered, as differences between pedigree and genomic rates of inbreeding were expected due to selection (Sonesson et al., 2012). A threshold for fitness was also imposed, and animals whose fitness was reduced by 50% or more were considered to be dead or unable to mate.

### Optimum Contribution Strategies

#### Maximize Genetic Gain (MGa and MGb Strategies)

The optimum contribution theory described by Meuwissen (1997) was used, adapted to genomic selection. The genetic gain in generation *t*+1 was defined by c_t_GEBV_t_, with c_t_ being the vector of contributions of selected candidates to generation *t*+1. This expression was maximized with Lagrange multipliers assuming a constraint for the average relationship of selection candidates $\overset{¯}{C_{t}}+1={c_{t}}^{\prime }G_{t}c_{t}/2=1-{\left(1-\Delta F_{G}\right)}^{t},$ where G_t_ was the genomic relationship matrix among selection candidates and Δ*F_G_* was the desired rate of genomic inbreeding (Sonesson et al., 2012), which was set to 0.01 or 0.005 in the present study. Once c_t_ was calculated, the offspring was produced by sampling a male and a female with replacement under random mating. Contributions were optimized for both sexes (MGa strategy) or only for sires (MGb strategy). These two strategies are considered relevant to current livestock genetic improvement program.

#### Minimize Rate of Inbreeding (MI Strategy)

The Lagrange multipliers’ approach was used to develop strategies relevant for a conservation program aiming to protect biodiversity by minimizing the rate of inbreeding for a given rate of allowed gain in the trait of interest. In this case, the constraint was set to ΔI + $\overset{¯}{I_{t}}$ , where $\overset{¯}{I_{t}}$ is the average index value observed for the population in generation *t* and Δ*I* is the desired rate of gain in the index (set to 0.30 in the present study). Contributions under this scheme were optimized for both sexes.

### Additional Considerations

Main scenarios tested are summarized in **Table 1.** Sires and dams selected in the optimum contribution scenarios described above were mated as dictated by the respective number of expected contributions of each selected parent assuming each mating produced a single offspring. Two mating strategies were tested in these main scenarios: under random mating, selected animals were mated at random with replacement. Under non-random mating, the static minimum kinship approach, which minimizes average co-ancestry (Ivy and Lacy, 2012) was followed. In the latter, individual mean molecular relationships (average molecular relationship of an animal with the rest of the population) were computed in every generation thereby creating two sex-specific lists where animals were ranked from lowest to highest mean relationship. The sire and dam with the lowest mean relationship were mated, followed by the sire and dam with the next lowest mean relationship. This process continued until all breeding pairs were formed. Any breeding pair with a relationship greater than the average relationship in the population was rejected, and the male was then mated to the next unpaired female with the lowest mean relationship.

**Table 1:** Summary of factor levels considered per strategy (MGa, MGb, MI, truncation of the best 10% of the animals and absence of artificial selection).

Strategy	Index	Const1 (%)	Const2	Propor (%)
MGa	I0/I25/I50	0.5/1.0	-	100/20
MGb	I0/I25/I50	0.5/1.0	-	100/20
Truncation selection	I0/I25/I50	-	-	100/20
MI	I0/I25/I50	-	0.3	100/20
Absence of artificial selection	-	-	-	-


*Factors considered were the proportion of fitness in the index (Index), the desired rate of genomic inbreeding (Const1) or genetic gain for fitness (Const2) and the proportion of animals with fitness phenotypic records (Propor).*

In addition to the main scenarios addressed in **Table 1**, other scenarios were simulated to test the concordance and validity of results. These secondary scenarios corresponded to (i) different number of males and females per generation (200 males and 800 females); (ii) different number of chromosomes (30 chromosomes to mimic the bovine genome); (iii) greater population size per generation (2,000 instead of 1,000).

### Scenario Assessment

All scenarios described above were run for 50 replicates and breeding strategies were compared for rates of inbreeding (Δ*F*), genetic gain (ΔTBV) and phenotypic change per generation. These rates were assessed in two intervals: from generation 0 to 5 (G0–G5) and from generation 6 to 20 (G6–G20), as it is expected that drift will also contribute to reducing the genetic variance in the early generations and, therefore, it may increase the early rates of inbreeding in the truncation methods. Rates of genomic inbreeding (Δ*F_G_*) were also compared with corresponding pedigree inbreeding rates (Δ*F_P_*) and were computed only for the last interval to avoid the first generations.

## Results

**Table 2** shows the results obtained from optimum contribution of both sexes, maximizing genetic gain for different inbreeding constraints (MGa). Results from truncation selection are included in **Table 2** for comparison. In all cases random mating among selected parents was assumed. Rates of genetic gain and genomic inbreeding under an MGa strategy with an inbreeding constraint of 0.01 (1%) were always similar to those obtained with the corresponding truncation selection scheme. However, a genomic inbreeding constraint of 0.005 (0.5%) in the MGa strategy reduced Δ*F_G_* by about half and the impact of inbreeding depression on fitness by 40–60%, while yielding only slightly smaller rates of genetic gain for the two traits (**Table 2**).

**Table 2:** Comparison of optimum contribution of both sexes for maximization of genetic gain (MGa) with the desired rate of genomic inbreeding (Const) and truncation selection (Truncation 10%) strategies under random mating: results are the observed rate of genomic inbreeding (Δ*F_G_*), the rates of genetic improvement (ΔTBV) in productivity and fitness and the rate of phenotypic change (Δ*P*) in fitness after accounting for inbreeding depression, for selection indices emphasizing 0, 25, and 50% on fitness (I0, I25, I50).

Trait	Strategy (Const)	Δ*F*_*G*_ (%)	Production (ΔTBV)		Fitness (ΔTBV)		Fitness (Δ*P*)	
					
		G6–G20	G0–G5	G6–G20	G0–G5	G6–G20	G0–G5	G6–G20
**Index = Production**								
I0	MGa (0.5%)	0.467	0.574	0.458	-0.291	-0.233	-0.410	-0.466
I0	MGa (1.0%)	0.896	0.662	0.478	-0.331	-0.240	-0.569	-0.693
I0	Truncation	0.950	0.637	0.479	-0.321	-0.243	-0.618	-0.721
**Index = Production + Fitness; All animals with fitness phenotypic records**								
I50	MGa (0.5%)	0.462	0.293	0.228	0.103	0.086	-0.010	-0.139
I50	MGa (1.0%)	0.887	0.337	0.233	0.115	0.091	-0.113	-0.350
I50	Truncation 10%	0.884	0.344	0.238	0.112	0.101	-0.188	-0.340
I25	MGa (0.5%)	0.467	0.522	0.419	-0.180	-0.151	-0.297	-0.381
I25	MGa (1.0%)	0.891	0.596	0.443	-0.196	-0.160	-0.427	-0.608
I25	Truncation	0.913	0.589	0.440	-0.200	-0.158	-0.506	-0.614
**Index = Production + Fitness; 20% of animals with fitness phenotypic records**								
I50	MGa (0.5%)	0.463	0.289	0.221	0.102	0.089	-0.012	-0.135
I50	MGa (1.0%)	0.885	0.333	0.233	0.117	0.092	-0.112	-0.350
I50	Truncation	0.865	0.334	0.235	0.113	0.097	-0.177	-0.334
I25	MGa (0.5%)	0.465	0.519	0.413	-0.170	-0.143	-0.287	-0.372
I25	MGa (1.0%)	0.890	0.591	0.434	-0.194	-0.156	-0.426	-0.605
I25	Truncation	0.908	0.588	0.439	-0.197	-0.158	-0.502	-0.613
**Average standard errors**								
-	MGa	0.002	0.005	0.003	0.004	0.002	0.004	0.003
-	Truncation	0.011	0.0048	0.0028	0.004	0.0026	0.0074	0.0062


**Table 3** shows the performance of an optimum contribution strategy to maximize the genetic gain applied only to males (MGb strategy) and considering random mating. When compared with optimum contribution of both sexes (**Table 2**), ΔTBV for productivity was generally reduced (30% on average) and ΔTBV for fitness was also reduced (26% on average) when productivity and fitness were equally weighted.

**Table 3:** Optimum contribution of sires for maximization of genetic gain (MGb) with the desired rate of genomic inbreeding (Const): results are the observed rate of genomic inbreeding (Δ*F_G_*), the rates of genetic improvement (Δ*TBV*) in productivity and fitness and the rate of phenotypic change (Δ*P*) in fitness after accounting for inbreeding depression, for selection indices emphasizing 0, 25, and 50% on fitness (I0, I25, I50).

Trait	Strategy (Const)	Δ*F*_*G*_ (%)	Production (ΔTBV)		Fitness (ΔTBV)		Fitness (Δ*P*)	
					
		G6–G20	G0–G5	G6–G20	G0–G5	G6–G20	G0–G5	G6–G20
**Index = Production**								
I0	MGb (0.5%)	0.474	0.382	0.347	-0.189	-0.178	-0.329	-0.413
I0	MGb (1.0%)	0.901	0.441	0.371	-0.217	-0.186	-0.495	-0.640
**Index = Production + Fitness; all animals with fitness phenotypic records**								
I50	MGb (0.5%)	0.472	0.203	0.176	0.066	0.063	-0.072	-0.168
I50	MGb (1.0%)	0.902	0.230	0.183	0.076	0.069	-0.197	-0.381
I25	MGb (0.5%)	0.470	0.351	0.316	-0.115	-0.110	-0.253	-0.342
I25	MGb (1.0%)	0.901	0.395	0.336	-0.131	-0.117	-0.404	-0.570
**Index = Production + Fitness; 20% of animals with fitness phenotypic records**								
I50	MGb (0.5%)	0.471	0.201	0.171	0.066	0.067	-0.070	-0.164
I50	MGb (1.0%)	0.901	0.227	0.184	0.074	0.070	-0.199	-0.382
I25	MGb (0.5%)	0.473	0.351	0.315	-0.117	-0.108	-0.255	-0.341
I25	MGb (1.0%)	0.903	0.397	0.340	-0.135	-0.121	-0.412	-0.574
**Average standard errors**								
-	MGb	0.002	0.004	0.003	0.003	0.002	0.004	0.003


The use of optimum contribution from a conservation perspective is exemplified in **Table 4**, which shows the results obtained under a strategy to minimize the rate of inbreeding for a given rate of genetic gain (MI strategy) under random mating. A scenario assuming absence of artificial selection is included in **Table 4** for comparison. Results suggest that the use of this strategy will lead to a rate of inbreeding similar to that observed in the complete absence of selection while at the same time allowing for a modest but noteworthy increase in genetic gain for productivity and fitness. The rate of phenotypic deterioration of fitness when compared to the absence of selection was reduced and, in some cases, halted and reversed.

**Table 4:** Optimum contribution of both sexes for minimization of inbreeding (MI) under random mating: results are the observed rate of genomic inbreeding (Δ*F_G_*), the rates of genetic improvement (Δ*TBV*) in productivity and fitness and the rate of phenotypic change (Δ*P*) in fitness after accounting for inbreeding depression and for selection indices emphasizing 0, 25, and 50% on fitness (I0, I25, I50); the constraint in the rate of gain for the index was 0.30.

Trait	Δ*F*_*G*_ (%)	Production (ΔTBV)		Fitness (ΔTBV)		Fitness (Δ*P*)	
				
	G6–G20	G0–G5	G6–G20	G0–G5	G6–G20	G0–G5	G6–G20
**Absence of artificial selection**							
-	0.049	0.001	-0.001	-0.001	0	-0.022	-0.017
**Index = Production**							
I0	0.016	0.151	0.143	-0.076	-0.072	-0.091	-0.078
**Index = Production + Fitness; all animals with fitness phenotypic records**							
I50	0.039	0.150	0.130	0.050	0.045	0.027	0.031
I25	0.022	0.191	0.176	-0.064	-0.061	-0.081	-0.069
**Index = Production + Fitness; 20% of animals with fitness phenotypic records**							
I50	0.038	0.144	0.127	0.047	0.043	0.026	0.030
I25	0.023	0.193	0.176	-0.067	-0.060	-0.084	-0.069
**Average standard errors**							
-	0.001	0.002	0.001	0.001	0.001	0.001	0.001


In scenarios with fitness records being available to a proportion of animals only, despite the lower accuracy, the rate of genetic gain decreased marginally (**Tables 1**–**3**), probably due to the combined effect of fitness (lower heritability) and productivity (higher heritability) and the relatively small difference in accuracies (0.1) among animals with and without genotypes.

**FIGURE 1:**
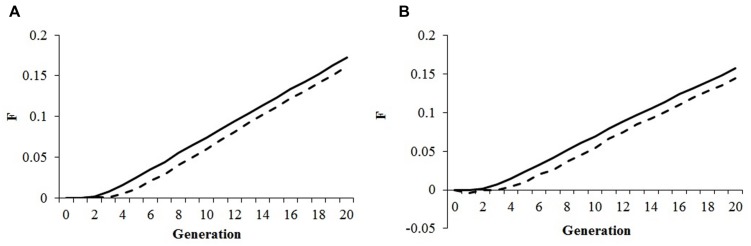
**Effect of mating strategy.** Pedigree inbreeding **(A)** and genomic inbreeding **(B)** in an optimal contribution of both sexes strategy to maximize genetic gain followed by either random mating (solid line) or mating based on the minimum kinship principle (dashed line); selection index was 50%/50% productivity/fitness; genomic inbreeding constraint was 0.01.

The use of an alternative mating program, based on optimum contribution followed by a mating scheme to minimize co-ancestry according to the minimum kinship method yielded interesting results that are summarized in **Figure 1.** The minimum kinship scheme minimized the average co-ancestry and inbreeding in the first four generations but afterward inbreeding rates increased to the same levels as under random mating of selected parents. This observation was independent of the genomic inbreeding constraint set for maximizing genetic gain, and applied equally to optimum contribution of both sexes and males only. No differences in the rates of genetic gain for productivity and fitness were observed when comparing minimum kinship with random mating (data not shown).

**FIGURE 2:**
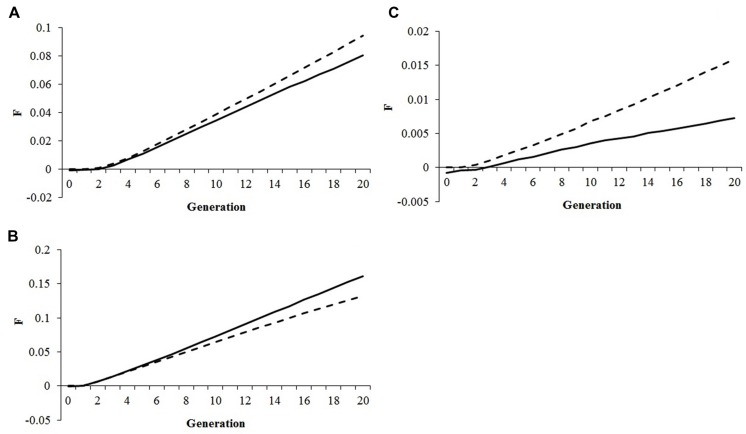
**Comparison between the rates of genomic (Δ*F_G_*, solid lines) and pedigree (Δ*F_P_*, dashed lines) inbreeding during the selection period.** **(A)** MGa with 50% selection emphasis on fitness assuming a constraint of 0.005 in the rate of genomic inbreeding and all animals having fitness records; **(B)** MI with 50% selection emphasis on fitness assuming a constraint of 0.30 in the rate of gain for the index and all animals having fitness records; **(C)** Truncation selection for the best 10% of animals with 50% selection emphasis on fitness.

A comparison between genomic and pedigree inbreeding rates is shown in **Figure 2.** Under optimum contribution and conservation approach, *ΔF_P_* overestimated *ΔF_G_* by 5–16% (**Figure 2A**) or by 30–69% (**Figure 2B**), whereas under truncation selection *ΔF_P_* underestimated *ΔF_G_* by 17–23% (**Figure 2C**). In absence of selection, no difference was observed between rates of pedigree and genomic inbreeding.

Additional analyses (**Supplementary Table S1**) led to very similar results when 30 chromosomes were simulated, instead of 20, in order to mimic the bovine genome. With the MGb strategy, use of a different number of males and females (200 and 800, respectively) in the population led to an approximate reduction of 16% in the rate of genetic gain for similar inbreeding levels when compared with equal number of sires and dams (500 each). In such case, the rate of increase in inbreeding with the MI strategy was nearly doubled. The effect of increasing the population size to 2,000 animals was more pronounced in the MI strategy, where the rate of inbreeding was reduced to about a third of that observed with 1,000 animals, whereas the rate of genetic gain in the MGb strategy slightly increased (∼5%) due to more selection opportunities.

## Discussion

The present study used a stochastic simulation to assess the performance of genomic-based optimum contribution strategies in animal breeding when dealing with production traits antagonistically related with fitness. Rates of genetic gain and phenotypic change per generation were assessed in two intervals as it is expected that selection will at first reduce the genetic variance, thus constraining the rates of genetic gain in early generations. Furthermore, the increased relatedness of selected individuals across generation will also impose a reduction in the rate of genetic gain when constraining the rate of inbreeding, leading to higher rates in the early periods of selection compared to later. Therefore, scenarios where strong selection is imposed (e.g., low restriction on genomic inbreeding) showed different rates between the two intervals, whereas scenarios with weak selection intensity (e.g., strict restriction on genomic inbreeding) led to similar rates of change in the two intervals.

Our results showed that the use of optimum contribution strategies with index selection can increase genetic gain for productivity and reduce (or even halt) the expected decay in fitness despite their antagonistic correlation. Optimum contribution may also alleviate the effect of inbreeding depression, even when the majority of animals cannot provide a phenotypic record for fitness. Compared with truncation selection, the use of optimum contribution will maintain a similar rate of increase in productivity while reducing the rate of inbreeding and the effect of inbreeding depression to one half. In addition, and considering a conservation perspective, our results showed that optimum contribution-based strategies (MI) can minimize inbreeding while maintaining or even improving other valuable traits such as production. These strategies would lead to a rate of inbreeding similar to that observed in absence of any selection, while at the same time yielding small but respectable increases in productivity and halting the decrease in fitness due to inbreeding depression.

The advantages of the use of genomic-based over phenotypic-based programs mainly depend on the accuracy of predicted breeding values and, therefore, the size of the training population. In our study we have assumed that the training populations (animals with phenotypes and genotypes) for both fitness and productivity are large enough to provide reasonable accuracies. Nevertheless, it is important to note that for traits with low heritability (fitness traits) the size of the training population needed to reach reasonable accuracies will be bigger than the one required for productivity traits. Daetwyler et al. (2010) showed accuracies for the validation set (animals with genotypes only) around 0.35 for traits with *h*^2^ = 0.1 and around 0.5 for traits with *h*^2^ = 0.3 when the training population had 1,000 individuals. Given these values and the extensive number of records generally available for cattle breeds, it is expected that reasonable accuracies as the ones considered in the present study would be reached. In the case of very small populations or breeds, the limited size of the training population would have an impact on the prediction accuracy, and thereby, reducing any potential benefit from combining genomic prediction with optimization of contributions. An extremely small training population may result in an accuracy too low to justify the use of genomic prediction alone and in combination with the optimization of contributions as suggested here.

In our study, the lack of fitness records in the majority of animals (80%) reduced the rate of genetic gain only marginally. This observation could be the result of a combined effect of (i) the drop in accuracy affecting only fitness and not productivity (as all animals have records for productivity); (ii) the small difference (0.1) between the accuracies for animals with and without fitness records; and (iii) the combination of two traits with different heritabilities and accuracies when creating the index. In order to clarify this situation, additional simulations (data not shown) considering the I50 scenario and a greater difference between fitness accuracies (0.5 for animals with records and 0.3 for animals without records) have shown higher reductions in genetic gain (∼8%), still leading to a reduced rate of inbreeding when compared with truncation selection.

In concordance with previous studies (Pryce et al., 2012; Sonesson et al., 2012; Clark et al., 2013), our results have shown a disparity between the rates of genomic (Δ*F_G_*) and pedigree (Δ*F_P_*) inbreeding during the selection period. Whereas the pedigree approach provides an expectation of the proportion of homozygosity in a given system, the molecular (genomic) approach using IBS reflects the true (realized) homozygosity. In absence of selection, both rates of inbreeding are similar, meaning that pedigree inbreeding is a good estimator of the realized inbreeding. However, the two measures of inbreeding differ when selection is applied, suggesting that the pedigree expectation may not be a good approximation of the realized genomic inbreeding. Therefore, and according to previous studies (Sonesson et al., 2012), it is recommended that a genomic-based selection scheme should consider genomic-based relationships among parents to control inbreeding, in order to derive more stable and predictable outcomes.

In conservation schemes, control of inbreeding can be additionally performed through the use of non-random mating systems without affecting the rate of genetic gain achieved for the trait of interest. Our results, in concordance with previous studies (Sonesson and Meuwissen, 2000; Fernández et al., 2011), showed that the use of the simplest mating strategy to minimize co-ancestry within a genomic-based program reduced the rate of inbreeding in the short term but did not prevent its subsequent increase, leading to a final rate of inbreeding consistent with the constraint applied at the optimum contribution step. Therefore, if mating strategies are expected to be used, it would be recommended to perform optimization of contributions and mating in a single-step in order to avoid implementation problems (Klieve et al., 1994; Fernandez et al., 2001; Kinghorn, 2011).

Based on our results, the use of optimum contribution strategies combined with genomic data appears to be a powerful tool to increase genetic gain while controlling inbreeding. However, before a large-scale implementation of these strategies, certain considerations need attention. Firstly, constraints in inbreeding and gain, and trait weights in the selection index have to be carefully considered. Secondly, strategies may be applied to one sex (i.e., males as in the MGb strategy in the present study) or both sexes (i.e., MGa and MI strategies). Consideration of both sexes will allow enhanced selection opportunities and thus a higher genetic gain for the same rate of inbreeding but, if the female reproductive rate is limited, the use of reproductive techniques (e.g., multiple ovulation, *in vitro* fertilization) will be necessary. Thirdly, the sex ratio of potential candidates will also have an impact on results depending on the chosen strategy and parameters. Under the MGb strategies presented here, the use of 200 males and 800 females led to a slight reduction in the rate of genetic gain for similar inbreeding levels when compared with 500 males and 500 females. Under an MI strategy, the effect was much stronger, leading to a twofold increase in the rate of inbreeding. Therefore, when working with an MI strategy, it would be recommended to consider a similar number of males and females to try to maximize the effective size. Of course each application should be tailored to the population structure relevant to the livestock species in question.

It is important to highlight two assumptions (based on the infinitesimal model) that were taken in the simulation to simplify the interpretation of results: first, inbreeding depression was simulated to be proportional to the average level of genomic inbreeding rather than as a function of the dominance effect and the loss of heterozygosity in the QTL (relative to the expected under Hardy-Weinberg equilibrium), thus making it dependent of the gene frequency. However, since our simulation assumed 1,000 QTLs with small effects, we expect that our approach would simulate comparable levels of inbreeding depression to the ones simulating dominance effects. Second, the approach used to calculate GEBVs means that accuracies were kept constant across generations, implying that the LD pattern between markers and QTLs is the same across the whole selection period. In practice, the LD patterns may change across generations and, thereby, the levels of accuracy of predicted GEBVs will also change. However, since all simulated scenarios were done using the same approach, the changes in LD would affect all cases similarly, therefore being the comparison across scenarios still valid.

Finally, and beyond the scopes of the present study, the use of genomic-based optimum contribution strategies in breeding programs has the additional advantage of measuring genomic IBS (or IBD) inbreeding only in specific chromosomal regions or genes of interest. This approach can, therefore, allow for a more precise control of homozygosity in specific regions related with fitness and/or rare alleles (Liu et al., 2014) or to minimize ROH (runs of homozygosity; regions of the genome where the copies inherited from our parents are identical) as proposed by Pryce et al. (2012). Studies are currently being performed to allow different inbreeding constraints for various chromosomal regions (Gómez-Romano et al., 2014).

## Conclusion

Our study demonstrated that the use of optimum contribution strategies in a genomic context effectively reduces the rate of increase in inbreeding while ensuring genetic improvement in traits of interest in a wide range of scenarios. The inbreeding impact on fitness was clearly contained, thus allowing the maintenance of fitness levels and, therefore, genomic-based optimum contribution strategies can be recommended both from conservation and animal genetic improvement perspectives.

## Author Contributions

ES-M participated in the study design, carried out the simulations and statistical analyses and drafted the manuscript. GB was responsible for the conception, funding, study design and implementation of the project. ES-M, RP-W, and GB managed the data analysis and manuscript preparation. All authors have read and approved the final manuscript.

## Conflict of Interest Statement

The authors declare that the research was conducted in the absence of any commercial or financial relationships that could be construed as a potential conflict of interest.
