# Supplementary material for: Genomic-Based Optimum Contribution in Conservation and Genetic Improvement Programs with Antagonistic Fitness and Productivity Traits
Source: Front Genet. 2016 Feb 24;7:25. doi: 10.3389/fgene.2016.00025 (PMC4764803; doi:10.3389/fgene.2016.00025)
Supplement: Supplementary file 1 [file Table_1.DOC]

Supplementary Material

Genomic–based optimum contribution in conservation and genetic improvement programs with antagonistic fitness and productivity traits

**Enrique Sánchez-Molano^1*^, Ricardo Pong-Wong^1^ and Georgios Banos^1, 2, 3^**

^1^ The Roslin Institute and Royal (Dick) School of Veterinary Studies, University of Edinburgh, Easter Bush, Midlothian EH25 9RG, Edinburgh, UK.

^2^ SRUC, The Roslin Institute Building, Easter Bush, Midlothian EH25 9RG, Edinburgh, UK.

^3^ School of Veterinary Medicine, Aristotle University of Thessaloniki, Greece.

***Correspondence:** Enrique Sánchez-Molano. The Roslin Institute and Royal (Dick) School of Veterinary Studies, University of Edinburgh, Easter Bush, Midlothian EH25 9RG, Edinburgh, UK. [Enrique.Sanchez-Molano@roslin.ed.ac.uk](mailto:Enrique.Sanchez-Molano@roslin.ed.ac.uk). +44(0) 131 6519221

# Supplementary Tables

**Supplementary Table 1. Additional scenarios comparing optimum contribution of sires for maximization of genetic gain (MGb) and minimization of inbreeding (MI) under different parameters such as number of animals (N animal), number of males (N males) and number of chromosomes (N chr). Results are the observed rate of genomic inbreeding (Δ*F_G_*), the rates of genetic improvement (Δ*TBV*) in productivity and fitness and the rate of phenotypic change (Δ*P*) in fitness after accounting for inbreeding depression. The selection index was always I50. The desired rate of genomic inbreeding for MGb strategy was 1%. The constraint in the rate of gain for the index was 0.30.**

| **N animal** | **N males** | **N chr** | **Trait** | **Strategy** | **Anim Phen (%)** | **ΔF_G_ (%)** | **Production (ΔTBV)** | | **Fitness (ΔTBV)** | | **Fitness (ΔP)** | |
| --- | --- | --- | --- | --- | --- | --- | --- | --- | --- | --- | --- | --- |
|  |  |  |  |  |  | **G6-G20** | **G0-G5** | **G6-G20** | **G0-G5** | **G6-G20** | **G0-**  **G5** | **G6-**  **G20** |
| 1000 | 200 | 20 | I50 | MGb | 100 | 0.894 | 0.195 | 0.165 | 0.061 | 0.059 | -0.212 | -0.391 |
| 1000 | 200 | 20 | I50 | MGb | 20 | 0.895 | 0.189 | 0.161 | 0.064 | 0.058 | -0.201 | -0.392 |
| 1000 | 200 | 20 | I50 | MI | 100 | 0.066 | 0.172 | 0.137 | 0.059 | 0.047 | 0.027 | 0.022 |
| 1000 | 200 | 20 | I50 | MI | 20 | 0.059 | 0.162 | 0.123 | 0.054 | 0.046 | 0.025 | 0.024 |
| 1000 | 500 | 30 | I50 | MGb | 100 | 0.896 | 0.232 | 0.193 | 0.084 | 0.071 | -0.183 | -0.381 |
| 1000 | 500 | 30 | I50 | MI | 100 | 0.038 | 0.148 | 0.132 | 0.052 | 0.046 | 0.029 | 0.030 |
| 2000 | 1000 | 20 | I50 | MGb | 100 | 0.891 | 0.249 | 0.199 | 0.099 | 0.070 | -0.173 | -0.376 |
| 2000 | 1000 | 20 | I50 | MI | 100 | 0.014 | 0.151 | 0.132 | 0.052 | 0.047 | 0.019 | 0.037 |
| **Average standard errors** | | | | | | | |  |  |  |  |  |
| - | - | - | - | - | - | 0.002 | 0.004 | 0.002 | 0.004 | 0.002 | 0.003 | 0.002 |
